# Confirmed presence of aedes (rusticoidus) *refiki* Medschid, 1928 in a continental dry Mediterranean peri-urban environment in south-central Spain

**DOI:** 10.1186/s40850-022-00124-x

**Published:** 2022-04-29

**Authors:** Laia Casades-Martí, Mario Frías, Sarah Delacour, Francisco Ruiz-Fons

**Affiliations:** 1 Health & Biotechnology (SaBio) Group, Instituto de Investigación en Recursos Cinegéticos, IREC (CSIC-UCLM-JCCM), Ciudad Real, Spain; 2 Infectious Diseases Unit, Instituto Maimonides de Investigación Biomédica de Córdoba (IMIBIC), Hospital Universitario Reina Sofía de Córdoba, Universidad de Córdoba, Córdoba, Spain; 3 CIBERINFEC, ISCIII - CIBER de Enfermedades Infecciosas, Instituto de Salud Carlos III, Madrid, Spain; 4 Departamento de Patología Animal, Facultad de Veterinaria, Universidad de Zaragoza, Zaragoza, Spain

**Keywords:** Aedes, Barcoding, Mediterranean, Peri-urban, Scarce

## Abstract

**Background:**

The ‘snow-melt mosquito’ aedes (*rusticoidus*) *refiki* is a rare species with a wide distribution in Europe that is usually defined as an aggressive mosquito for mammals, including humans. During a mosquito survey in a peri-urban area in south-central mainland Spain, adult *Ae. refiki* females were captured and identified by morphological traits. The presence of this species of mosquito has never been molecularly confirmed under continental dry Mediterranean climatic influence with scarce number of days with snow on soil. The aim of this study was to confirm by amplification and sequencing of mitochondrial cytochrome c oxidase subunit I (COI) and internal transcribed spacer 2 (ITS2) region.

**Results:**

We also successfully amplified and typed the species molecularly by COI and ITS2 regions. The peri-urban area where *Ae. refiki* was found contrasts with the reported cold, humid and snowy environments required by the species to breed.

**Conclusions:**

This finding suggests that the species is already adapted to continental dry Mediterranean environments, questioning whether it is a truly stenotopic species of cold snowy environments.

## Background

Mosquitoes are known as vectors of numerous widely distributed diseases, such as Dengue, Chikungunya, Yellow Fever, Zika, West Nile, or malaria, among others. To date, more than 3,500 species of these Diptera are known worldwide, many of them with a relevant epidemiological role due to their vectorial competence [1–4].

**Fig. 1:**
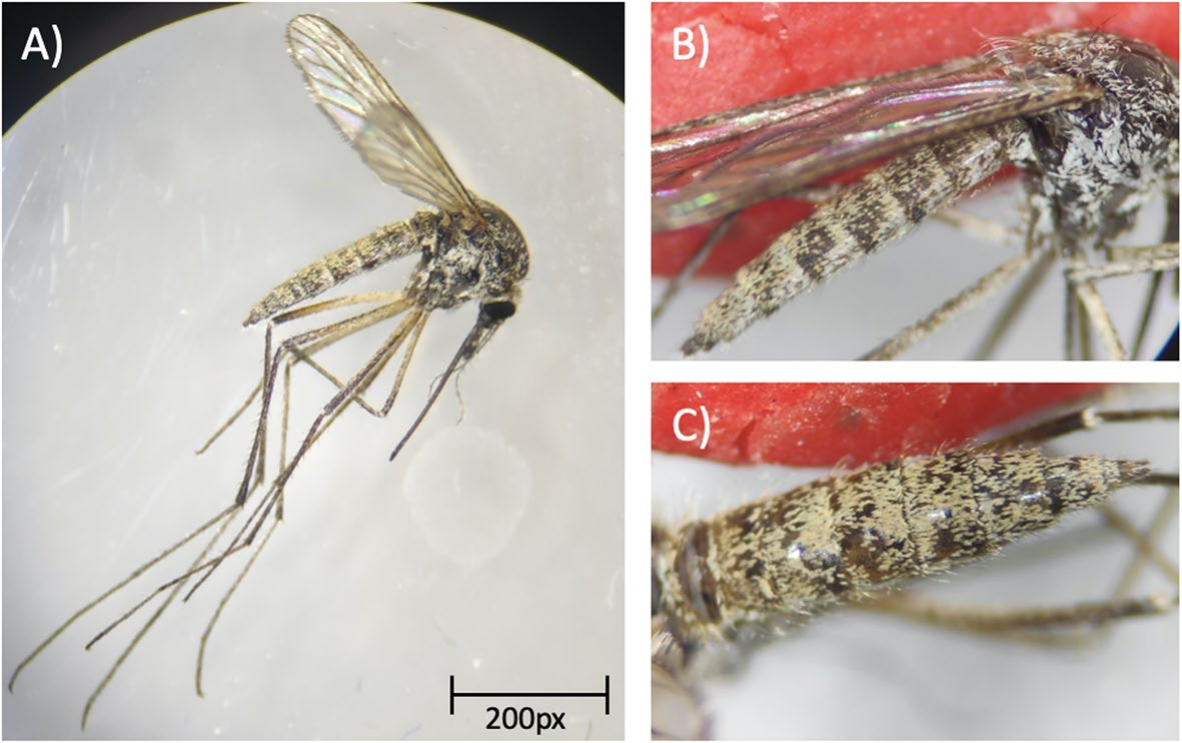
Images under magnification (Leica S9D, Leica microsystems, Germany) of aedes refiki. **A** Right lateral view of a complete female. **B** Magnified right lateral view of the abdominal area, showing the scattered pale scales forming apical bands. **C** Enlarged dorsal view of the terga showing the scattered pale scales and the absence of the median longitudinal band

After the introduction and establishment or permanent populations of aedes* albopictus* and aedes* japonicus* [5, 6], sixty-five species of mosquitoes can be found in Spain [7]. Their distribution is variable depending on adaptability and environmental conditions, although there are some species that are widely distributed throughout almost the entire Iberian Peninsula (IP), such as *Ochlerotatus caspius*, *Culex theileri, Cx. pipiens* or *Culiseta longiareolata* [8]. However, information on other species is scarce, such as aedes* (rusticoidus) refiki*, a rare species known as a ‘snow-melt mosquito’ that is usually found in very limited or specific areas where low temperatures and altitudes above 1,000 m predominate [9, 10].

aedes* refiki* was first described by Medschid, 1928 [11], who included it in the aedine subgenus *rusticoidus* Shevchenki & Prudkina, 1973, according to Reinert [12], and to Wilkerson & Linton [13]. This species occurs across Europe, from the Iberian Peninsula in the West to Anatolia in the East, and from Sweden in the North to Italy in the South [10]. In Spain, the first description dates back to 1944 in the east-centre of the mainland country [14]. Some years later (1979 and 1982), the species was reported in the west-centre of the country [15, 16], and the last report of the species in the IP consisted in the collection of *Ae. refiki* larvae in the Iberian System, in Guadalajara and Teruel provinces in east-central Spain [17]. Morphologically, *Ae. refiki* closely resembles *Oc. rusticus*, but it differs in terga colouration (Fig. 1) [10, 18]*. Aedes refiki* is a considered to be a stenotopic species commonly detected in areas with altitudes between 1,000 and 2,000 m associated with cold environments [10, 14, 17, 19–23]. Their biology is very similar to that of *Oc. rusticus*, ovipositing in semi-permanent freshwater areas with a neutral-alkaline pH [9, 10, 17, 24, 25]. Aedes* refiki* is a monocyclic species [14, 16, 19, 24, 26]. It spends the winter in the egg form, and the larvae, which are able to survive in extreme cold conditions, hatch at the beginning of the year during ice/snow melting [20]. The first adults appear between late April and early May, the adult flight period occurring between May and June. Aedes* refiki* females are mammophilic and feed mainly at dusk, but they can also feed during the day [10, 18, 24, 27] causing a serious seasonal ectoparasitic problem for humans and animals [19]. It is not yet known whether they are vectors of specific pathogens [27].

Aedes* refiki* does not fly long distances but tend to stay in shaded areas close to breeding sites. Therefore, their locations are often very local [10]. Consequently, both population estimates and the distribution of the species may be underestimated. Given this fact, we should not rule out the potential role of this species in disease transmission, as is the case with other species of the same genus [4, 28]. Therefore, for the establishment of known distribution patterns, studies that molecularly confirm the morphological identification of this species are necessary.

This study reports the first occurrence of *Ae. refiki* in an area with environmental conditions strongly deviate from those usually described for the species. Moreover, since its identification in Spain has only been done by morphological traits, the aim of this work was also to confirm molecularly the identification of *Ae. refiki* in the IP, as this will facilitate future studies on its possible ability to adapt to different environmental conditions.

## Results

**Fig. 2:**
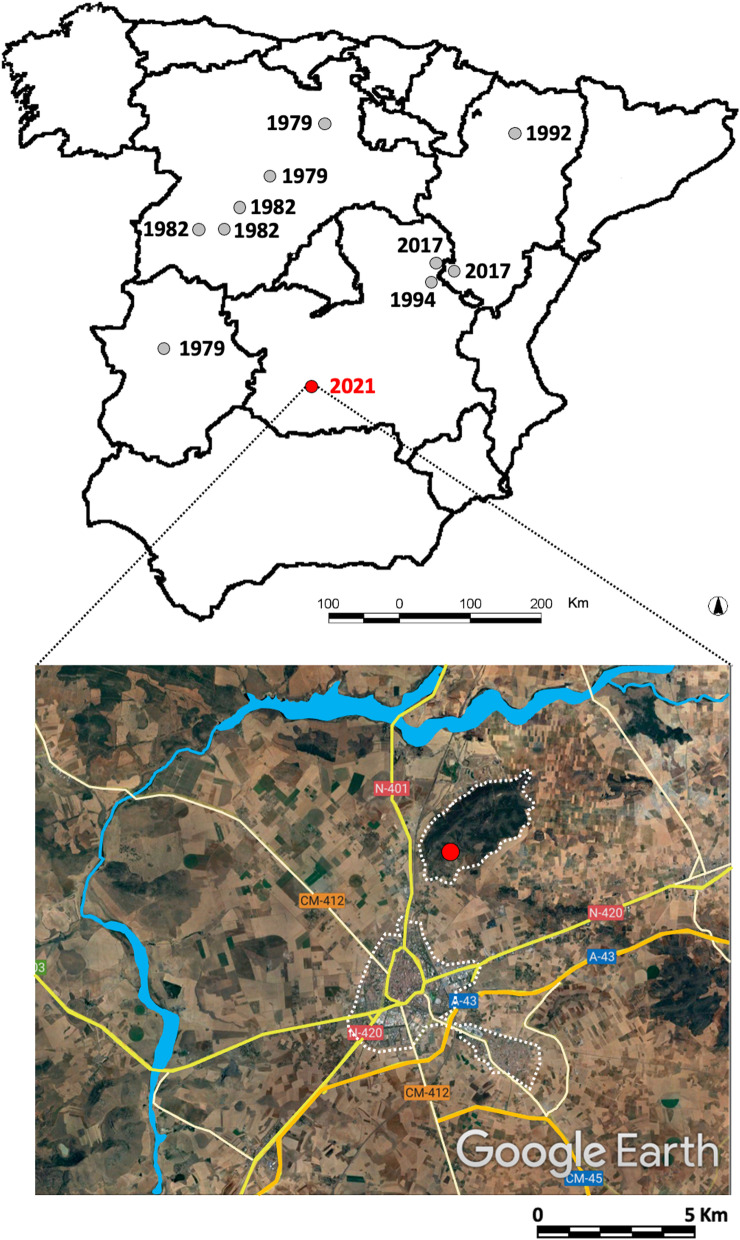
Map of Spain that summarizes where aedes* refiki* has been described. The red dot indicates the location of collection of the aedes* refiki* mosquitoes in this study. The grey-shaded dots represent the locations where the mosquitoes have been detected before in Spain (report year shown close to dots). The white dashed lines correspond to the urban areas of Ciudad Real, Miguelturra and La Atalaya (location of the study). The roads are marked in yellow and orange depending on their size (motorway or regional road). The nearby wetlands are highlighted in blue (Guadiana river and the Vicario reservoir). This image is freely available and has been modified by the authors using the Microsoft PowerPoint programme

The morphological identification revealed that 17 of the adult mosquitoes captured in the trap were females of the species *Ae. refiki* according to the broad black scales on the upper postpronotum, and by the terga with scattered pale scales forming basal and apical bands, with no median longitudinal line (Fig. 1). The mtDNA sequences (COI) of 9 of them and the rDNA sequences (ITS2) of 10 of them were finally analysed because of available sequences. The former matched previously described gene sequences (BLAST) for *Ae. refiki*. Alignment of the sequences with GenBank base entries resulted in 97% identity with sequences described by Kuhlisch et al. [18] in Germany. Figure 2 shows a phylogenetic tree where the sequences obtained in this study are compared with the COI strains described in Genbank for this species. The ITS2 sequences had not been described for this species so far.

## Discussion

This study confirms the detection of *Ae. refiki* in May under unusual weather conditions and altitude according to existing reports in western Europe. Studies carried out previously in Castilla-La Mancha have never detected *Ae. refiki* or morphologically similar species before (The authors, unpublished results). The few old existing records of *Ae. refiki* suggest that it is a rare species and that it frequents areas where low temperatures predominate. Thus, many authors [10, 21–22] describe *Ae. refiki* as a ‘snow-melt’ mosquito, resistant to extreme cold conditions and capable of breeding even in snowy areas at high altitude, taking advantage of snow melting water to develop at larval stages. Rueda et al. [17] detected larvae of this species in the Iberian System (240 km from our study area) in areas above 1,500 masl and with an average annual temperature of 10.5 °C (AEMET; Period: 1981–2010. Altitude: 1,062 masl. Latitude: 40° 50′ 30'' N, Longitude: 1° 52′ 44'' W). Kuhlisch et al. [18] additionally provided a detailed history of the patchy historic records of *Ae. refiki* throughout Germany at altitudes ranging from the sea level up to 500 masl, although in Oceanic influenced temperate and humid continental climates with cold snowy winters. However, it remains debatable whether this species could be thermophilic or thermotolerant, since already in 1971 and 1982 some specimens were found at altitudes below 1,000 m in France and west-central Spain [16, 21], and also in hot coastal plains (0-200 m altitude) with scarce rainfall in western Turkey [26]. According to our findings and to the sporadic reports of the species in environmentally dry areas of Europe, *Ae*. *refiki* could be a plastic species, able to survive in diverse environments of the southern Spanish Plateau. The presence of the species in the reported environment was confirmed by molecular barcoding, providing no doubt of this new report in the study area. We can therefore assure that the meteorological conditions and altitude of this record do not match those described as specific for the species [10, 21, 22], suggesting that it might not be as stenotopic as previously thought [19].

Bueno-Marí et al. [7] sampled areas with favourable conditions for the species in which they did not detect its presence. However, considering that this species is univoltine, and that adults emerge between April and May [10, 16, 21, 27], the timing of sampling should be considered to detect the species. It should be noted that existing reports on *Ae. refiki* in Spain have so far based the identification of adults and larvae on morphological characters. Therefore, considering the great similarity of these species with other species such as *Oc. rusticus*, identification errors could have occurred. This study confirms for the first time using molecular tools the presence of *Ae. refiki* in Mediterranean environments, being so far from the southernmost area of the IP with positive findings of this species. The presence of permanent water sources in the peri-urban environment where *Ae. refiki* was captured in this study, such as swimming pools, a disused water reservoir or irrigation ponds, may have favoured the establishment of the species in an apparently marginally optimal environment.

Although Rueda et al. [17], did not report attacks or bites despite detecting the presence of this species in the early morning hours, Kuhlisch et al. [18] described it as an annoying mosquito. This agrees with the fact that during the setting of the traps of our study we observed highly aggressive mosquitoes, confirming that *Ae. refiki* seems to be an aggressive biter. Furthermore, Schaffner et al. [27] and Becker et al. [10] describe it as a mammalophilic species that frequents shaded areas where it may bite humans and mammals even during the day. In this sense, and according to our finding, it would be convenient to take into account the probable adaptability of *Ae. refiki* to warm and continental environments. Although *Ae. refiki* has not been to date proven as relevant in the transmission of pathogens [10, 27], its potential vector role for numerous mosquito-borne pathogens should not be ruled out [2, 4]. Furthermore, extemporaneous sampling should be carried out in all types of habitats and environmental conditions to further clarify the geographic range of the species in Mediterranean environments.

## Conclusions

This study demonstrates the presence of *Ae. refiki* in hotter and drier climates than those usually reported for the species, as well as at lower altitudes than those described as characteristic for the species. In the peri-urban study area the absence of pristine waters is noteworthy, showing a high adaptability of *Ae. refiki* to diverse breeding environments, and a possible predilection for areas with a strong urban influence and a notable human presence. The genetic sequence obtained coincides with those already described so far, being the first time that it has been contrasted in the IP.

## Methods

**Fig. 3:**
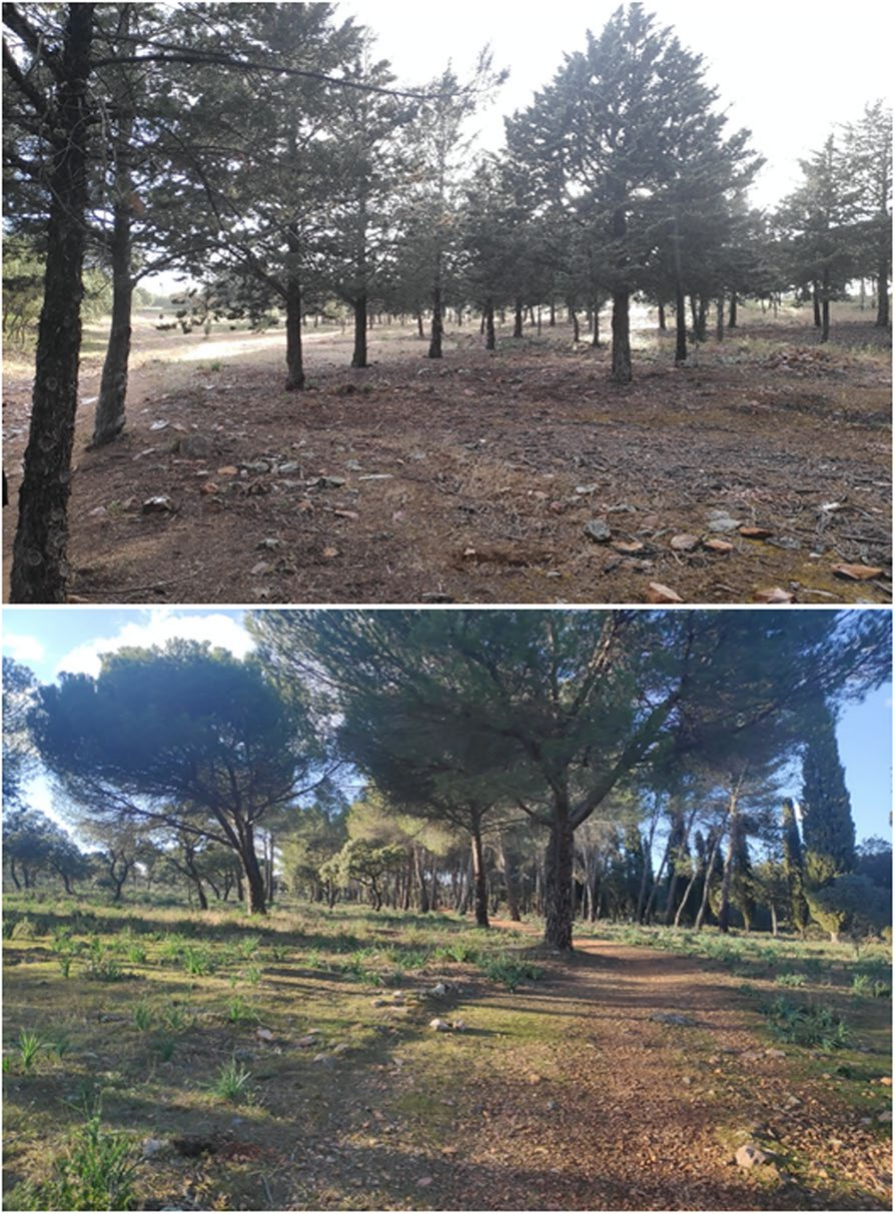
Photos obtained in March 2022 from the area where the mosquito collection was carried out. (La Atalaya park, Ciudad Real province, Spain.)

**Fig. 4:**
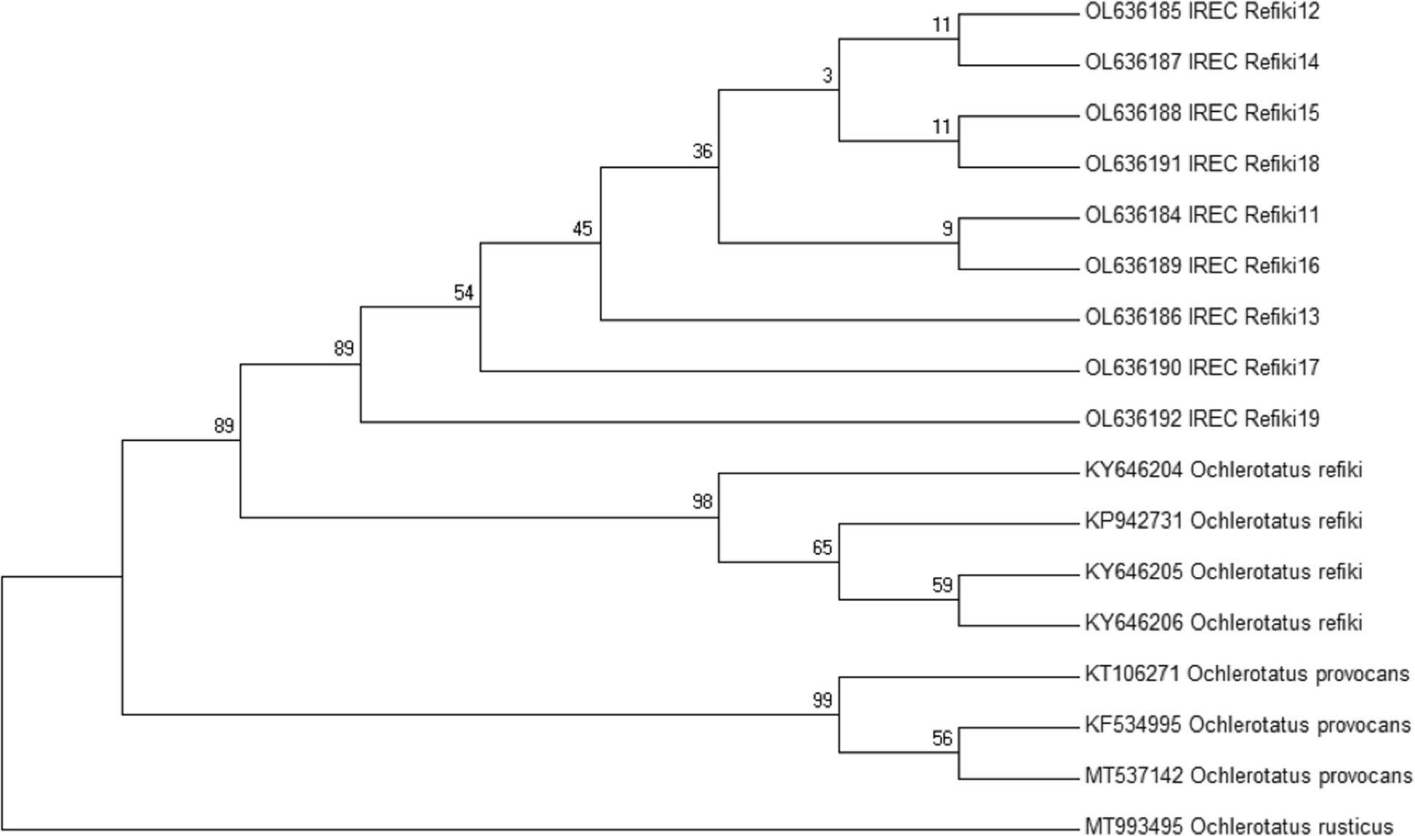
Philogenetic tree performed with COI sequences described in this study. The evolutionary history was inferred by using the Maximum Likelihood method based on the Tamura-Nei model. The bootstrap consensus tree inferred from 1000 replicates is taken to represent the evolutionary history of the taxa analyzed. Branches corresponding to partitions reproduced in less than 50% bootstrap replicates are collapsed. Initial tree(s) for the heuristic search were obtained by applying the Neighbor-Joining method to a matrix of pairwise distances estimated using the Maximum Composite Likelihood (MCL) approach. The analysis involved 17 nucleotide sequences. All positions with less than 95% site coverage were eliminated. There were a total of 423 positions in the final dataset. Evolutionary analyses were conducted in MEGA6 [30]

In May 2021, mosquitoes were collected in a peri-urban area (the forest park of ‘La Atalaya’; 39°01′39.6"N 3°55′10.2"W) located at an Euclidean distance of 3 km from the limits of the city of Ciudad Real (Spain) (Fig. 3 and 4). This is a highly human-influenced Mediterranean ecosystem with a mixture of shrubs (*Retama* spp., *Quercus faginea*, *Cystus ladanifer*, *Pistacia lentiscus, Stipa gigantea*), grasslands, autochthonous holm oak (*Quercus ilex*), and human-introduced pinelands (*Pinus halepensis* and *P. pinaster*), scattered cypress (Cupressaceae) and eucalyptus (Myrtaceae) trees. The south-western slope of the hill where the forest park is located is an urbanised residential area with a large number of recreational swimming pools and irrigated gardens. The altitude ranges 620–704 masl. The annual average rainfall is 402 mm and the average annual temperature is 15.6 °C. Average winter, spring, summer and autumn temperatures are 8.5 °C, 18.0 °C, 24.8 °C and 10.9 °C, respectively. In addition, the average annual number of snow days is 2.6, and snow has only been observed between December and April (Spanish Meteorological Agency, AEMET; www.aemet.es). Snow does only cover the soil for few days every 5–10 years. Two centimetres of snow fell on the area on January 7, 2021, but snow melted in less than 24 h with rain. Climate series data were freely accessed at the Spanish Meteorological Agency (AEMET) website (http://www.aemet.es/en/) and refer to the period 1981–2010. The altitude of the meteorological station is 628 masl and it is located in latitude 38° 59′ 21'' N, longitude: 3° 55′ 13'' W.

For mosquito collection, two CDC (Centers for Disease Control and Prevention) white light traps baited with dry ice as a CO_2_ source were hanged from a tree at a 1.7 m height in the survey area during one night. Diptera retained in the collection canister were transferred to the laboratory and stored at -80 oC until analysis.

The morphological identification of the mosquitoes was performed using the keys provided by Becker et al. [10] with a magnifying stereoscopic microscope (Leica S9D, Leica microsystems, Germany). In the laboratory, the abdomens of mosquitoes morphologically resembling *Ae. refiki* were processed to obtain the sequences of COI (mitochondrial cytochrome c oxidase subunit I) and the ITS2 (internal transcribed spacer 2) region for barcoding analyses. Mosquito DNA was purified using Nucleospin® DNA Insect kit (Macherey–Nagel, Düren, Germany), following the manufacturer's instructions. For COI (629 bp), a PCR was performed using primers LCO1490 (5'-GGTCAACAAATCATAAAGATATTGG-3') and HCO2198 (5'-TAAACTTCAGGGTGACCAAAAAATCA-3') based on study by Folmer et al. [23]. The PCR reaction was carried out using the Master Mix PCR kit (Promega, Madison, USA). The 50 μl PCR reaction consisted of 12.5 μl of extracted DNA, and the PCR reaction conditions were as follows: an initial denaturation of 94 °C for 2 min, followed by the amplification reaction for 40 cycles of denaturation at 94 °C for 30 s, annealing at 50 °C for 45 s and extension at 72 °C for 45 s, followed by a final extension at 72 °C for 10 min. For ITS2 (435 bp), primers ITS2 5.8F (5′-TGTGAACTGCAGACACACATG-3′) and ITS2 28R (5′-ATGCTTAAATTTAGGGGGGGTA-3′) were used [29]. The same volumes and concentrations of reagents were used for PCR as for COI PCR assay. Thermal cycler conditions were 94 °C 1 min denaturation, pre-amplification of 5 cycles at 94 °C 1 min, 45 °C 1.5 min and 72 °C 1.5 min, followed by 35 cycles of 94 °C 1 min, 57 °C 1.5 min and 72 °C 1 min and a final extension step at 72 °C 5 min. Gel purification of the amplicons was performed for COI (QIAquick®, Gel Extraction Kit, Qiagen, Germany) whereas for the ITS amplicons, the purification was directly performed from the PCR product (QIAquick®, PCR Purification Kit, Qiagen, Germany). The purified samples were sent for Sanger sequencing to Eurofins Genomic GmbH (Ebersberg, Germany). The sequences obtained were compared using the NCBI BLAST tool (Megablast, nucleotide collection (nr/nt) database) with GenBank sequences available for *Ae. refiki* (KP942731, KY646204, KY646205 and KY646206), *Ochlerotatus provocans* (KF534995, KT106271 and MT537142) and *Oc. rusticus* (MT993495). The sequences obtained in this study are available at GenBank under accession numbers OL636184, OL636185, OL636186, OL636187, OL636188, OL636189, OL636190, OL636191, OL636192 for COI and OL639696, OL639697, OL639698, OL639699, OL639700, OL639701, OL639702, OL639703, OL639704, OL639705 for ITS2.

## Data Availability

All data generated or analysed during this study are included in this published article [and its supplementary information files].

## Abbreviations

COI: Mitochondrial cytochrome c oxidase subunit I
CDC: Centers for disease control and prevention
ITS2: Internal transcribed spacer 2
IP: Iberian peninsula
Masl: Meters above sea level
PCR: Polymerase chain reaction
